# Pet Insurance and Veterinary Treatment Decisions: A Game-Theoretic Model of the Owner–Veterinarian Relationship in Türkiye

**DOI:** 10.3390/vetsci13101005

**Published:** 2026-09-23

**Authors:** Betül Zehra Gençgönül

**Affiliations:** Department of Actuarial Sciences, Hacettepe University, 06800 Ankara, Türkiye; betul.zehra@hacettepe.edu.tr

**Keywords:** treatment-acceptance threshold, utility function, Nash equilibrium, game theory, pet insurance, veterinarian-owner relationship

## Abstract

The economic benefits of pet insurance are generally examined through survey-based empirical studies, while a game-theoretic explanation of how insurance changes treatment decisions remains limited. This study models the clinical decision process between a pet owner and a veterinarian as a static, two-player game within expected utility theory, using an exponential (constant absolute risk aversion, CARA) utility function. The veterinarian’s strategy is {Basic, Comprehensive Treatment}, the owner’s is {Accept, Reject}; severity is a two-state random variable (Mild/Severe). Parameters are calibrated using Turkish market data from the author’s previous study. Findings show that in the uninsured regime, a unique Nash equilibrium (Basic Treatment, Reject) endogenously produces economic treatment-refusal behavior; insurance, by altering the payoff structure, makes available a second, Pareto-superior equilibrium (Comprehensive Treatment, Accept). Sensitivity analyses show comprehensive treatment becomes relatively more attractive as risk aversion increases, but once the co-payment rate exceeds a threshold (~46.3%), insurance loses this effect entirely. Premium level does not affect equilibrium selection, being a sunk cost independent of the treatment decision. These findings indicate that the co-payment rate is a more critical policy variable than premium level in insurance design, and offer a game-theoretic explanation for how pet insurance may affect the veterinarian–owner treatment decision.

## Simple Summary

Pet owners may delay or refuse treatment for a sick animal because they cannot afford the cost. This study models the interaction between a pet owner and a veterinarian as a simple decision game to examine how insurance may affect treatment decisions. It calculates, mathematically, how the choices of the owner and the veterinarian compare under insured and uninsured conditions. Under the baseline assumptions, treatment rejection emerges as the owner’s dominant strategy in the uninsured regime, whereas insurance makes an additional Pareto-superior treatment-acceptance equilibrium available. However, this effect is not unlimited; once the share of treatment costs paid out of pocket exceeds a model-implied threshold, the additional equilibrium is no longer available. The baseline co-payment rate used in the model is a descriptive benchmark based on selected pet insurance products publicly offered in Türkiye, rather than a representative market-wide estimate. These results illustrate how the owner’s out-of-pocket share may influence treatment decisions and provide a theoretical framework for the further empirical investigation of pet insurance and veterinary care.

## 1. Introduction

The literature on the economic and welfare effects of pet insurance relies largely on survey-based empirical studies. However, few studies offer a micro-founded, game-theoretic explanation of why insurance changes treatment decisions. The clinical decision process between an owner and a veterinarian is, in fact, a strategic interaction between two interdependent parties: the veterinarian’s recommendation shapes the owner’s decision, and the owner’s expected response shapes the veterinarian’s recommendation. This mutual dependence is the classic unit of analysis in game theory.

Indeed, Williams et al. [1], using survey data collected from dog owners in the United States, applied multiple regression models (OLS, negative binomial, multinomial logit) and found that pet health insurance ownership has a significant, positive effect on veterinary expenditures. Verteramo Chiu et al. [2] examined the factors influencing insurance demand among uninsured pet owners using econometric methods. Boller et al. [3], through a retrospective cohort study, showed that insured owners of dogs diagnosed with gastric dilatation-volvulus chose presurgical euthanasia at a significantly lower rate than uninsured owners. Kipperman et al. [4] surveyed veterinarians’ views on the effects of cost of care and economic constraints on patient outcomes. In addition, Volk et al. [5], through a large-scale survey (the Bayer Veterinary Care Usage Study), documented pet owners’ veterinary care usage patterns in the United States with descriptive statistics; Lue et al. [6] examined the effect of the owner–pet bond and the owner–veterinarian relationship on the quality of care received; Springer et al. [7] comparatively assessed veterinarians’ attitudes toward pet insurance in Austria, Denmark, and the United Kingdom through a survey; and Wilson [8] provided a descriptive account of the underwriting criteria and practices of pet health insurance companies in the United States. A common feature of these studies is that they measure the effect of insurance indirectly, through observed outcomes (expenditure level, euthanasia rate, demand, attitude); the strategic mechanism through which insurance changes the decision process between the veterinarian and the owner is not directly modeled.

From a veterinary practice perspective, the benefits of insurance are not limited to owner expenditures. Dunn [9] discussed the role of third-party payment systems, including insurance, in the billing processes and financial sustainability of small animal practice, noting that insurance contributes to practice stability by making clinics’ cash flow more predictable. Coe et al. [10], through a focus group study, examined veterinarians’ and owners’ perceptions of the monetary aspects of care, showing that communication difficulties around cost directly affect the clinical decision process—a finding that directly parallels the owner–veterinarian strategic interaction modeled in this study. Becker et al. [11] discussed the potential of insurance to improve veterinary care within an ethical framework, and also drew attention to its limits. In addition, insurance databases constitute a valuable resource for veterinary epidemiology; Egenvall et al. [12] examined the distribution of morbidity and mortality in an insured dog population in Sweden, showing how insurance records can be used for large-scale animal health surveillance.

It is also known that game theory and agency theory (principal agent) frameworks are used in strategic decision modeling in animal health economics. A comprehensive scoping review by Hennessey et al. [13] identified 31 studies in this area, but found that most focused on livestock disease control and biosecurity decisions. An example also exists in the companion animal context: Sykes and Rychtář [14] modeled cat owners’ decisions to vaccinate against toxoplasmosis using game theory, showing a critical cost threshold beyond which owners abandon vaccination.

In contrast to this literature, we treat the effect of insurance not as an indirect observed outcome relationship or a one-sided vaccination decision, but as a two-player game involving the mutual strategic interaction of the veterinarian and the pet owner. The model calculates the utility levels of both the owner and the veterinarian separately under insured and uninsured scenarios, comparing the Nash equilibria that balance—or fail to balance—the utility of both parties. This approach not only describes the potential effect of insurance on treatment decisions, but also identifies how changes in the payoff structure make an additional treatment acceptance equilibrium feasible.

Game theory and expected utility theory provide a well-established mathematical framework for studying decision-making under uncertainty. In insurance economics, the classic expected utility framework explains why risk-averse parties prefer to substitute an uncertain loss for a certain, smaller cost by paying a premium [15,16]. In this study, the same framework is applied to the veterinarian–owner interaction, providing a theoretical mechanism for how insurance may change treatment decisions.

The Turkish companion animal population is characterized by substantial geographical and socio-demographic heterogeneity, which is relevant when interpreting the economic and veterinary implications of pet insurance. Previous estimates used in the present modeling framework indicate approximately 4.7 million domestic cats and 1.4 million domestic dogs in Türkiye, while national identification records have provided an important basis for estimating the owned companion animal population [17]. Recent nationwide survey evidence further indicates that pet owners’ age, education, income level, and place of residence are associated with differences in animal ownership, care practices, and knowledge, suggesting that the economic capacity and demographic profile of owners may influence veterinary decision-making [18]. Breed composition also appears to be heterogeneous. In a study of Turkish dog owners, Özcan et al. [19] reported that breed selection was associated with adaptation ability, social influence, expected working function, and breed-specific characteristics; gender, education level, housing type, number of children, ownership of other pets, and the owner’s level of professional involvement with dogs were also associated with breed choice patterns. More recent evidence from different geographical regions of Türkiye identified Kangal Shepherd, German Shepherd, Golden Retriever, Pomeranian, and Toy Poodle among the breeds examined in owned-dog populations, with Kangal representing an important indigenous breed and the latter four representing commonly encountered non-indigenous companion breeds [20]. For cats, a recent survey of 1000 owners in Ankara reported that 51.6% of cats were tabby (Tekir), followed by British (13.0%), Scottish (10.2%), and Van cats (4.0%), indicating a predominance of non-pedigree cats alongside several locally and internationally recognized breeds [21]. These findings suggest that the Turkish companion animal population should not be considered homogeneous with respect to either owner characteristics or breed composition. Moreover, research using the Turkish adaptation of the Cat/Dog Owner Relationship Scale indicates that owner–dog relationships encompass emotional closeness, interaction, and perceived cost, with perceived cost varying according to educational status [22]. Although breed-specific owner–animal relationships have not been sufficiently characterized across Türkiye, available evidence suggests that both owner characteristics and breed-related attributes may shape acquisition preferences and human–animal interactions. Because the present study does not analyze a primary empirical sample of owners or animals, these findings are used as contextual evidence rather than as study-specific sample characteristics. Accordingly, these contextual factors should be considered when interpreting the present theoretical model and when generalizing its implications to the broader Turkish pet insurance market.

To fill this gap, the present study models the interaction between the pet owner and the veterinarian as a static, complete-information, two-player game, and examines the strategic behaviors of both parties within expected utility theory [23,24]. The role of insurance is treated as an exogenous mechanism that alters the game’s payoff structure; in this way, the potential effect of insurance on treatment decisions is examined not merely as an empirical observation, but through the changes in the game’s equilibrium structure induced by insurance. The model is calibrated with premium, coverage, and loss ratio data obtained for the Turkish pet insurance market in the author’s previous study, grounding the findings in the Turkish context [17].

## 2. Materials and Methods

### 2.1. Game Theory: Conceptual Framework and Rationale

Game theory is a discipline that mathematically models the strategic interaction of rational decision-makers (players) who are mutually interdependent. This approach, systematically formalized by Von Neumann and Morgenstern [24], analyzes situations in which each player, while trying to maximize their own utility, also takes into account the other players’ possible strategies. Nash [23] defined an equilibrium concept (the Nash equilibrium) in which no player can increase their utility by unilaterally deviating from their strategy, while the other’s strategy remains fixed; since then, this concept has become a fundamental tool of decision analysis in many disciplines, particularly economics, biology, and health economics.

A natural extension of game theory in health economics is the analysis of insurance markets. Arrow [15] examined the fact that medical care is consumed under uncertainty, and how this uncertainty is managed through insurance mechanisms; Borch [16] addressed the risk-sharing problem within a formal game-theoretic equilibrium framework. These studies show that insurance is not merely a financial product, but also a mechanism that reshapes the strategic interaction between parties (e.g., moral hazard, adverse selection).

There are three main reasons for choosing game theory in this study. First, the veterinarian–owner interaction is inherently interdependent; the veterinarian’s recommendation affects the owner’s decision, and the owner’s expected response affects the veterinarian’s recommendation—this is the classic unit of analysis in game theory. Second, explaining the effect of insurance not merely through descriptive statistics (e.g., increased spending) but through the underlying changes in the game’s equilibrium structure offers a theoretical mechanism that may help explain why insurance works—offering more generalizable implications for policymakers and insurers. Third, although applications of game theory in animal health economics are increasing [13], these applications are predominantly focused on livestock disease control decisions; the only example in the companion animal (pet) context, the study by Sykes and Rychtář [14], addresses a vaccination decision rather than insurance. A game-theoretic model of the owner–veterinarian interaction in an insurance context does not, to our knowledge, exist in the literature; indeed, Hennessey et al. [13] specifically highlight that strategic decisions such as insurance adoption in the companion animal sector remain an unexplored area. This study aims to fill that gap.

### 2.2. Game Setup

In this study, the interaction between the two rational decision-makers facing each other in the veterinary clinical setting is formulated as a static (simultaneous-move), complete-information, finite-strategy game.

• Players: Pet Owner (Policyholder, S) and Veterinarian (V)

Veterinarian’s strategy set: t ∈ {T, K}—Recommend Basic Treatment (T) or Comprehensive Treatment (K).

• Owner’s strategy set: a ∈ {Accept, Reject}—Accept or reject/delay the recommendation.

The animal’s true clinical severity s ∈ {L, H} (Mild/Severe) is modeled as an exogenous, random state of nature; the probability distribution Pr(s = H) = p, Pr(s = L) = 1 − p is taken as common knowledge for both players. This represents a risky portfolio structure qualitatively consistent with the 60–70% loss ratio reported by Gençgönül [17].

Neither player observes the realized severity s prior to choosing their strategy. The prior probability p is treated as common knowledge, reflecting the residual diagnostic uncertainty that characterizes many presenting complaints in veterinary practice even after clinical examination—for instance, cases in which the definitive severity of a condition is confirmed only once treatment is underway, once diagnostic test results become available, or as the disease progresses. Both the veterinarian’s recommendation and the owner’s response are accordingly chosen ex ante, under the common prior p, and the realized state s subsequently determines the realized outcome; this is why payoffs are expressed as certainty equivalents (Section 2.3) rather than as state-contingent strategies.

We formulate the interaction as a static, simultaneous-move game rather than a sequential (Stackelberg) game for three reasons. First, the model’s primary objective is to characterize which strategy combinations are mutually self-enforcing—that is, Nash equilibria from which neither party can profitably deviate unilaterally—rather than to represent the literal temporal order of a clinical encounter; this is a standard simplification when the analytical focus is equilibrium characterization rather than sequential rationality. Second, because neither player observes the realized severity state before acting, the veterinarian’s recommendation cannot itself be conditioned on private information about the true state, which reduces the strategic value that moving first would otherwise confer. Third, veterinary treatment recommendations in practice are often shaped by standardized clinical protocols and professional guidelines established prior to any individual encounter, rather than strategically tailored in real time to an anticipated owner response, which is more consistent with a simultaneous, protocol-based formulation than with a fully sequential one. We nonetheless acknowledge that an explicitly sequential formulation, in which the veterinarian moves first and the owner responds after observing the recommendation, would more closely mirror the temporal structure of a clinical visit; we return to this point in the Limitations and in the Conclusions.

In the present model, ‘Basic Treatment’ and ‘Comprehensive Treatment’ represent two stylized levels of treatment intensity rather than universally appropriate clinical choices. Comprehensive Treatment is intended to represent a higher-intensity treatment package that may be clinically warranted in severe cases, whereas Basic Treatment represents a lower-intensity option. The model does not assume that Comprehensive Treatment is clinically superior in every disease state; in particular, it does not explicitly model the potential welfare cost of unnecessary treatment in mild cases. Accordingly, the terms should be interpreted as economic treatment categories used to represent different treatment costs and expected welfare consequences, rather than as a clinical ranking of treatment quality.

### 2.3. Utility Function: Constant Absolute Risk Aversion (CARA)

Both players are assumed to be risk-averse, with increasing-concave utility functions—the classic expected utility condition u′(w) > 0, u″(w) < 0 [25]. In this study, an exponential (CARA) utility function is chosen for analytical tractability and because the Arrow–Pratt risk-aversion coefficient is independent of (constant across) wealth level,

(1) *v*(*w*) = −*e*^−^*^αw^*       (Owner),        *u*(*w*) = −*e*^−^*^γw^* (Veterinarian)

where α, γ > 0 are the parties’ absolute risk-aversion coefficients, and $r(w)=-\frac{u\prime \prime (w)}{u\prime (w)}=\alpha$ (constant). This property makes it unnecessary to incorporate the players’ initial wealth (w0) into the model; only the net wealth change (Δ) resulting from the decision is relevant.

Under the two-state (Mild/Severe) random loss structure, the utility of each strategy combination (cell) is expressed in terms of the certainty equivalent (CE),

(2) $$\mathrm{CE}(t,a)=-\frac{1}{\alpha }\cdot \ln[(1-p)\cdot e^{-\alpha \cdot \Delta_{L(t,a)}}+p\cdot e^{-\alpha \cdot \Delta_{H(t,a)}}]$$

Applied to the owner, this yields *CE_S_*(*t*,*a*) using the risk-aversion coefficient α; applied to the veterinarian, it yields *CE_V_*(*t*,*a*) using γ. This representation makes the payoff matrix directly interpretable in TRY, providing an intuitive presentation for the reader.

### 2.4. Net Wealth Change Functions

The owner’s net wealth change is defined, depending on the insurance regime (Uninsured/Insured), the treatment decision, and the realized state, as follows:

(3) Δ*_S_*(*t*,*a* = *Accept*,*s*) = −*P* · 1[*Insured*] − *λC_t_* − *ψ_H_* · 1[*t* = *T*,*s* = *H*]

(4) Δ*_S_*(*t*,*a* = *Reject*,*s*) = −*P* · 1[*Insured*] − *ψ_s_*

Here λ ∈ [0, 1] represents the share of the treatment cost the owner pays out of pocket—that is, the co-payment rate in the insurance policy; λCt is the portion of the treatment cost paid out of pocket by the owner, while (1 − λ)Ct is the portion covered by the insurer (λ = 0 means full insurance coverage, λ = 1 means uninsured/full payment). In the uninsured regime, λ = 1 (full payment) is assumed. The term ψ_H_·1[t = T, s = H] represents the undertreatment welfare loss arising when only basic treatment is administered in a severe case.

The veterinarian’s net income, being deterministic and independent of the realized state, depends only on their own recommendation and the owner’s response.

Clinical appropriateness is not separately parameterized in the veterinarian’s utility function. Instead, the present model abstracts from clinical efficacy and represents the veterinarian’s payoff through treatment revenue, operating costs, and the reputational/ethical cost associated with treatment refusal. Consequently, a higher veterinarian payoff from Comprehensive Treatment should not be interpreted as evidence that this treatment is clinically superior.

(5) Δ*_V_*(*t*,*a* = *Accept*) = *R_t_* − *η_t_*        Δ*_V_*(*t*,*a* = *Reject*) = −*κ_t_*

where *R_t_* is treatment revenue, *η_t_* is operating cost, and *κ_t_* is the reputational/ethical cost incurred upon refusal.

Because Δ*_V_*(*t*,*a*) does not depend on the realized state s, the veterinarian faces no outcome-level uncertainty conditional on the strategy pair (*t*,*a*), and the realized payoff is known with certainty once (*t*,*a*) is fixed. Consequently, under the present specification, the veterinarian’s risk-aversion coefficient γ does not affect the certainty equivalent *CE_V_*(*t*,*a*) = Δ*_V_*(*t*,*a*), nor does it influence equilibrium selection; the veterinarian’s utility function u(w) is retained for structural symmetry with the owner’s problem and to allow for natural extensions in which the veterinarian’s payoff is itself rendered stochastic—for example, by incorporating complication risk, delayed insurer reimbursement, or malpractice exposure (see Limitations).

### 2.5. Model Parameters

Model parameters are derived from two different sources: (i) directly observed market data, and (ii) reasonable assumptions made to illustrate the model’s qualitative equilibrium mechanism.

Treatment costs (Ct, Ck) and the annual premium (P) are directly calibrated using Turkish pet insurance market data analyzed by Gençgönül [17] (Table 1). The baseline co-payment rate (λ = 20%) is used as a descriptive benchmark based on publicly available co-payment rates reported for selected pet insurance products in Türkiye. Specifically, Akbank’s Pati Plus product reports a 20% insured co-payment at contracted veterinarians [26]; the Allianz Sevimli Dostum product page reports 30% for Mini, Midi, and Maxi plans and 20% for the Premium plan [27]; Olâ’s Vet Amerikan Plans 1, 2, and 3 report 10%, 20%, and 10%, respectively [28]; Fiba Sigorta’s Fi-Pati products cover 85% of eligible treatment expenses, implying a 15% owner share [29]; and Türkiye Sigorta’s Sempati Pet Insurance applies a 20% co-payment at contracted veterinary institutions and a 30% co-payment when services are obtained from non-contracted institutions [30]. The simple arithmetic average of these eleven listed product/plan rates is approximately 21.36%, which is close to the 20% level; therefore, 20% is used as the baseline descriptive benchmark in the model. This figure should be interpreted as a descriptive benchmark based on selected publicly available products, rather than as a statistically representative estimate of the Turkish pet insurance market.

The probability of a severe case (*p* = 0.35) is not itself a directly observed market parameter; it is an illustrative value motivated only qualitatively by the 60–70% loss ratio reported in [17], since an aggregate portfolio loss ratio and the probability that an individual clinical case is severe are conceptually distinct quantities. We adopt *p* = 0.35 for demonstration purposes and separately examine its influence on the results (see sensitivity analysis in Section 3 and Limitations). The veterinarian’s revenue and operating cost parameters (Rt, ηt) are derived based on the typical profit margin range of treatment cost (approximately 75–80%).

By contrast, ψ (the welfare cost of refusal) and κ (the veterinarian’s reputational/ethical cost) do not have directly observable market values; they are therefore monetized assumptively. This approach relies on the “willingness-to-pay” logic used in health economics to incorporate non-monetary outcomes (pain, risk of loss, psychological distress) into decision models. Specifically, ψ_H_ (15,000 TRY) is set to represent the weight of the likely consequences of refusing treatment in a severe case at a magnitude close to, but below, the comprehensive treatment cost (CK = 25,000 TRY)—this ordering (ψ_H_ < C_K_) is a necessary condition for the model to produce economic treatment-refusal behavior, a pattern in the veterinary literature associated with, and in its most extreme form termed, “economic euthanasia” when refusal results in the animal’s death [3]. ψ_L_ (500 TRY) is set on the assumption that refusal in a mild case corresponds to a relatively small cost. κ_T_ and κ_K_ (1000 and 6000 TRY, respectively) monetize the veterinarian’s experience of “moral distress”; the assumption κ_K_ > κ_T_ rests on the premise that the refusal of a more serious clinical recommendation imposes a greater ethical burden on the veterinarian. These four parameters are derived not from empirical measurement but from reasonable assumptions capable of illustrating the model’s qualitative equilibrium mechanism.

Similarly, the value α = 0.0003 used for the owner’s risk-aversion coefficient in the baseline scenario (Table 1) also stems from an illustrative choice rather than an empirical estimate; this value was set to produce a risk premium pronounced enough to make the model’s mechanism observable, yet moderate enough not to assume excessive risk aversion. The sensitivity of the results to this choice is systematically tested in Section 3.2, where α is varied over a wide range (0 to 0.0013).

### 2.6. Equilibrium Analysis

A 2 × 2 payoff matrix was constructed for each regime; pure-strategy Nash equilibria were identified through standard best response analysis. To test the robustness of the model, a sensitivity analysis was carried out across the parameters not directly calibrated using market data: (i) the owner’s risk-aversion coefficient (α), (ii) the probability of a severe case (p), (iii) the co-payment rate (λ), (iv) the premium level (P), (v) the welfare cost of refusal (ψH), and (vi) the veterinarian’s reputational cost (κK). For (ii), (v), and (vi), we additionally recompute the co-payment threshold λ* introduced in Section 3.3 to assess how sensitive its numerical value is to these illustrative parameters (Table 2). All computations were performed using Python 3.12, with NumPy 2.4 and Matplotlib 3.10 for visualization).

The analysis focuses on pure-strategy Nash equilibria because the study is primarily concerned with stable combinations of discrete treatment recommendations and owner responses that have a direct clinical interpretation. Mixed-strategy equilibria were not the focus of the present analysis, as probabilistic randomization over treatment recommendations or treatment acceptance is less directly interpretable in the clinical setting. This restriction does not imply that mixed-strategy equilibria cannot exist; rather, they are beyond the scope of the present equilibrium comparison.

## 3. Results

### 3.1. Baseline Equilibrium Results

Figure 1 presents, comparatively for both regimes, the certainty equivalent (CE) payoff matrices calculated under the exponential (CARA) utility function. In the uninsured regime, the game has a single pure-strategy Nash equilibrium: (Basic Recommendation, Reject). In this equilibrium, the owner’s CE is −11,580 TRY, and the veterinarian’s CE is −1000 TRY. Notably, for the owner, the “Reject” strategy is dominant regardless of the treatment type recommended by the veterinarian; CE falls to −25,000 TRY if comprehensive treatment is accepted, and to −19,569 TRY if basic treatment is accepted—in both cases worse than the Reject option (−11,580 TRY). This result shows that the model, in an uninsured setting, endogenously produces economic treatment-refusal behavior; the owner is driven to refuse treatment out of cost concerns, independently of the animal’s welfare.

In the insured regime, the structure of the game changes fundamentally. While the old equilibrium (Basic, Reject) persists, a second, Pareto-superior equilibrium emerges—(Comprehensive Treatment, Accept), where the owner’s CE rises to −9250 TRY (an improvement of +6580 TRY relative to the old equilibrium), and the veterinarian’s CE rises from −1000 TRY to +11,000 TRY. This welfare comparison of the two equilibria shows that the Pareto-superior equilibrium in the insured regime strictly dominates the uninsured equilibrium for both the owner and the veterinarian.

This result illustrates a broader structural feature of the game; introducing insurance changes it from a setting with a single, treatment-refusal equilibrium into a coordination game with two equilibria, only one of which is Pareto-superior. The model does not itself specify which equilibrium will be selected in the insured regime, and the fact that the original (Basic, Reject) equilibrium persists alongside the new one means that the availability of the Pareto-superior outcome does not guarantee its realization. We discuss this equilibrium-selection problem, and possible real-world coordination mechanisms such as trust and communication between veterinarian and owner, in the Discussion.

### 3.2. Sensitivity to Risk Aversion

Figure 2 shows how the CE values of the three main strategies change as the owner’s risk-aversion coefficient (α) increases. Because the Comprehensive Treatment + Accept option has a deterministic outcome (treatment cost does not depend on the realized state), its CE value remains constant, independent of α. In contrast, the Reject and Basic + Accept options involve random outcomes depending on the realized state (s), so their CE values decrease as α increases due to the risk premium. As a result, risk-averse owners find comprehensive treatment relatively more attractive—fully consistent with the classic insurance economics proposition that insurance is especially valuable for risk-averse individuals, and emerging here as an endogenous result of the model rather than an external assumption.

**Figure 2:**
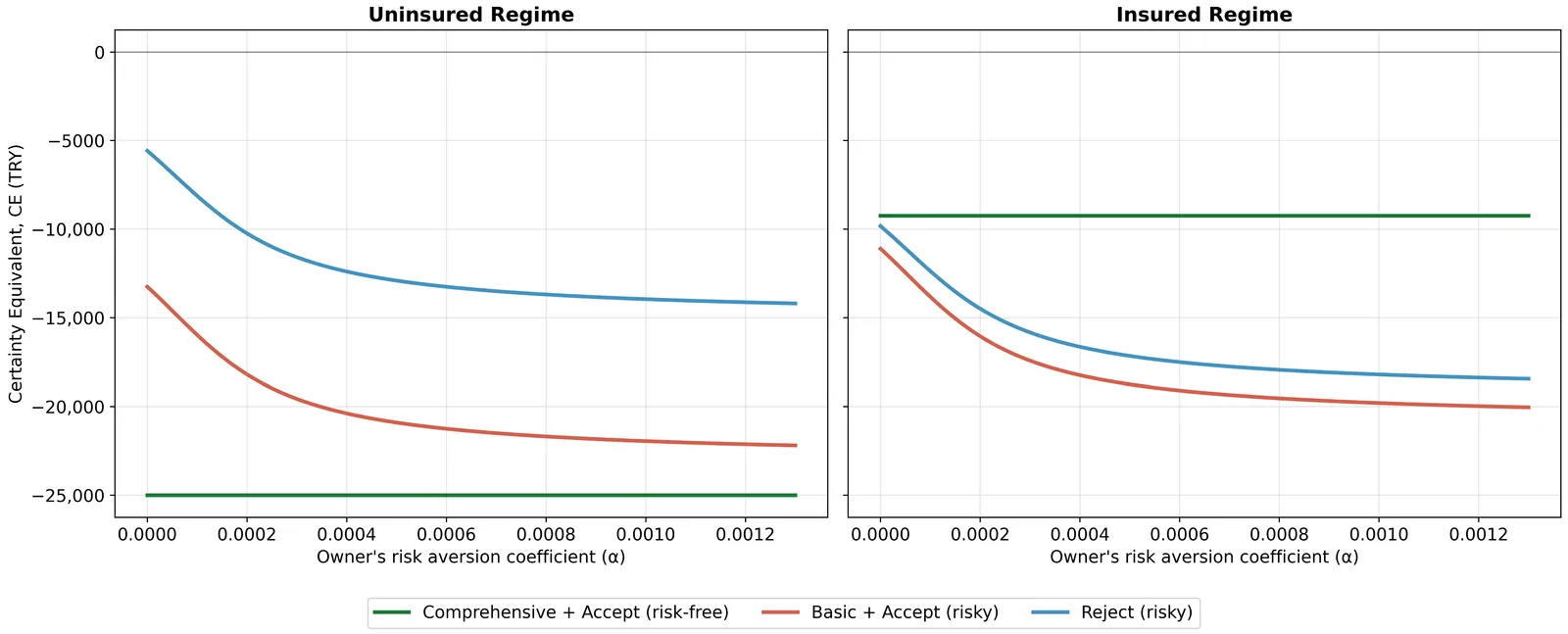
Effect of risk aversion on the relative attractiveness of comprehensive treatment.

### 3.3. The Treatment-Acceptance Threshold: The Critical Role of the Co-Payment Rate

Our strongest policy-relevant finding concerns the co-payment rate (λ). Figure 3 shows that as λ increases, the CE value of the Comprehensive + Accept equilibrium decreases linearly, whereas the CE value of the Reject option is unaffected by λ (constant at −15,830 TRY, since the premium payment is present equally under both options, and the co-payment comes into play only when treatment is accepted). The two curves intersect at λ* ≈ 46.3%. Below this threshold, insurance keeps the Pareto-superior equilibrium sustainable; above it, the Pareto-superior equilibrium disappears entirely and the game reverts to the single (Basic, Reject) equilibrium—insurance becomes effectively non-functional once the owner’s out-of-pocket share is too high. The selected publicly available Turkish pet insurance products considered in Section 2.5 report co-payment rates ranging from 10% to 30%, with the observed rates concentrated at 10%, 15%, 20%, and 30%. The simple arithmetic average of these eleven listed product/plan rates is approximately 21.36%, which is close to the 20% level; therefore, 20% is used as the baseline descriptive benchmark in the model [26,27,28,29,30]. These observed product-level rates are below the model-implied threshold, but this comparison should not be interpreted as evidence that the broader Turkish market lies below the threshold, because the benchmark is based on selected products and the threshold itself depends on illustrative model parameters (see Limitations).

To assess the robustness of this threshold, we examined its sensitivity to parameters not directly calibrated from market data. Table 2 reports λ* under variation in the severe-case probability (p), the welfare cost of refusal (ψ_H_), and the veterinarian’s reputational cost (κ_K_), holding other parameters at baseline. Across the tested range of p (0.20–0.80), the Pareto-superior equilibrium remains available at the default co-payment rate (λ = 20%) in every case, and λ* itself increases with p (from 39.3% to 57.1%), indicating that a higher probability of severe cases, if anything, widens rather than narrows the range of co-payment rates over which insurance remains effective. By contrast, λ* is considerably more sensitive to ψ_H_, ranging from 27.4% to 66.2% across a ±33% variation in this illustrative parameter; this confirms that the numerical value of the threshold—though not its qualitative existence—depends materially on this assumed parameter, and should accordingly be interpreted as a model-implied benchmark under the stated assumptions, rather than a precisely estimated policy target. λ* is, however, entirely insensitive to κ_K_ across the tested range (3000–9000 TRY), because the threshold is determined by the owner’s indifference condition between Accept and Reject, which does not depend on the veterinarian’s payoff parameters.

Figure 4 shows how this threshold forms a “feasibility region” in premium–co-payment space. A theoretically notable result of the model is that this threshold is independent of the premium level (P); mathematically, the premium cancels out of the equation because it enters with equal weight in both compared cells (K-Accept and T-Reject). In other words, the premium is a sunk cost that does not affect the owner’s decision at the moment of treatment; only the marginal co-payment rate determines the outcome. The three premium scenarios reported by Gençgönül [17] (3500/4250/5000 TRY) all fall within this feasibility region at the baseline λ = 20%; this is a model-based comparison and should not be interpreted as evidence that these scenarios represent the entire Turkish pet insurance market.

## 4. Discussion

An important qualification concerns equilibrium selection. Because the original (Basic, Reject) equilibrium persists alongside the new, Pareto-superior equilibrium once insurance is introduced, the model establishes that a better outcome becomes available but does not, on its own, determine which equilibrium will be realized in practice. We hypothesize that real-world coordination mechanisms—such as mutual trust between veterinarian and owner, effective communication about treatment options and costs, and the veterinarian’s willingness to recommend comprehensive care—may influence which equilibrium is reached; this is a plausible extension of the formal analysis rather than a result derived from the model itself, and it would benefit from direct empirical investigation in future work.

This study complements the macroeconomic/actuarial findings previously reported by Gençgönül [17] with a micro-founded strategic-interaction model. Whereas that earlier study demonstrated the potential market size and technical profitability of pet insurance in Türkiye, the present study offers a theoretical mechanism that may help explain why insurance improves treatment acceptance rates and the veterinarian–owner relationship; insurance, by altering the payoff structure, changes the game from a single-equilibrium “avoidance regime” to a multi-equilibrium “cooperation regime.”

Our findings are consistent with observational studies in the international literature. The empirical finding that insured owners accept recommended treatments at a higher rate [5,6] is theoretically consistent with our model, in which insurance makes an additional Pareto-superior equilibrium (Comprehensive Treatment, Accept), available alongside the original (Basic, Reject) equilibrium. Because the model does not specify an equilibrium-selection mechanism, this result should be interpreted as identifying an additional feasible treatment-acceptance outcome rather than predicting a necessary shift from one equilibrium to the other. Similarly, the empirical finding that insurance reduces the incidence of presurgical euthanasia [3] is theoretically consistent with the present model. In the uninsured regime, Reject is the owner’s dominant strategy, and this treatment-refusal mechanism may provide a possible pathway through which financial constraints can contribute to treatment denial in practice. However, euthanasia is not explicitly modeled as an action or outcome in the present framework; therefore, the model does not establish a direct causal link between insurance and euthanasia. Rather, the result provides a theoretical mechanism that may be relevant to the relationship documented in the empirical literature.

The treatment-acceptance threshold identified in this study relates to an important debate in the veterinary insurance literature regarding moral hazard [8]. Moral hazard is typically framed as an overuse problem from the insurer’s perspective; our model points to a related but distinct mechanism, in which—under the baseline parameterization—a co-payment rate set too high can undermine the insurance’s own purpose of increasing access to treatment. This points to a model-implied threshold value (λ ≈ 46.3% under baseline assumptions) that may be of interest to insurance product designers—meaning that “light” policies that combine a low premium with a high co-payment, while appearing affordable, may in practice lack the power to change treatment decisions. As several of the underlying parameters are illustrative rather than empirically estimated (see Limitations), this threshold should be interpreted as a model-implied benchmark under the stated assumptions, rather than as a validated, generally applicable policy target.

The finding that the premium level does not affect equilibrium selection at the treatment stage, although counterintuitive at first glance, is conditional on insurance having already been purchased; the model does not address the separate decision of whether to purchase or renew a policy, in which the premium plausibly plays a central role. Within this treatment-stage scope, the result suggests that co-payment structure deserves at least as much attention as premium level in policy design—a point that may also be relevant to the comprehensive information campaigns recommended by Gençgönül [17] for helping owners compare policies.

From the veterinarians’ perspective, the findings show that insurance broadens not only owner behavior but also clinical decision-making freedom; in the insured Pareto-superior equilibrium, the veterinarian’s CE (+11,000 TRY) is markedly higher than even their best outcome in the uninsured regime (+4000 TRY, Basic + Accept). This is consistent with the broader literature [1,4,7] suggesting that insurance may reduce the extent to which veterinarians’ recommendations are constrained by owners’ ability to pay; within the present model, this manifests specifically as increased owner willingness to accept the veterinarian’s comprehensive treatment recommendation, rather than as evidence that the model itself identifies which treatment is clinically optimal in a given case.

### Limitations

Several limitations of this study should be noted. First, several parameters—the welfare cost of refusal (ψ), the veterinarian’s reputational cost (κ), and the owner’s risk-aversion coefficient (α)—are illustrative assumptions rather than empirically estimated values (Table 1); consequently, the numerical results reported here, including the approximately 46.3% co-payment threshold, should be interpreted as model-implied benchmarks under the stated assumptions, rather than as precisely estimated policy parameters. Second, the probability of a severe case (*p* = 0.35) is only qualitatively motivated by the aggregate insurance loss ratio reported in [17], and is not itself a directly calibrated market parameter (Section 2.5); although our sensitivity analysis (Table 2) shows the qualitative results are robust to this parameter, more precise calibration would require case-level clinical data. Third, the game is formulated as a static, simultaneous-move interaction, whereas the clinical encounter it represents is inherently sequential, with the veterinarian typically recommending treatment before the owner responds (Section 2.2); a sequential (Stackelberg) formulation may yield different equilibrium predictions and is a natural direction for future work. Fourth, the model assumes complete information, including common knowledge of the probability distribution over disease severity; real clinical interactions may instead involve asymmetric information between veterinarian and owner regarding both the animal’s condition and the owner’s financial constraints. Fifth, disease severity is represented by only two states (Mild/Severe); this simplification does not capture the continuous nature of clinical severity in practice. Because the study does not use a primary empirical sample, study-specific socio-economic characteristics or breed frequencies cannot be reported as sample descriptors; the Turkish contextual evidence presented in the Introduction is therefore intended to inform external validity rather than to characterize the model as representative of a particular sampled population.

Sixth, the veterinarian’s payoff function does not include an explicit clinical-appropriateness component; comprehensive treatment is modeled as financially preferable to the veterinarian in the relevant equilibrium, but this need not imply that comprehensive treatment is clinically optimal in every realized state, and the model does not penalize potentially unnecessary treatment intensity in mild cases. Seventh, the insurance company is not modeled as a strategic player; premium-setting, underwriting, and reimbursement policies are treated as exogenous parameters rather than as outcomes of a three-way strategic interaction. Eighth, the model addresses only the treatment-stage decision conditional on insurance already having been purchased; it does not model the owner’s decision to purchase or renew a policy, and the finding that the premium is inconsequential for treatment-stage equilibrium selection should not be extended to claims about the overall importance of premiums in insurance uptake or product design. Ninth, and relatedly, the numerical co-payment threshold reported in this study has limited external validity beyond the specific parameterization examined here; it should be understood as illustrating a qualitative mechanism—the existence of a point beyond which cost-sharing undermines insurance’s ability to support treatment acceptance—rather than as a validated estimate applicable to the Turkish market, or to other markets, as a whole.

## 5. Conclusions

This study examined the effect of pet insurance on the veterinarian–owner interaction within a static game theory model and an exponential (CARA) utility function framework. The findings show that in an uninsured setting, refusing treatment emerges as the dominant strategy, providing a game-theoretic basis for economic treatment refusal, a pattern related to what the literature terms “economic euthanasia” when it results in the animal’s death. The entry of insurance into the market, by altering the payoff structure, makes an additional Pareto-superior equilibrium (acceptance of comprehensive treatment) available alongside the original (Basic, Reject) equilibrium. Rather than implying a necessary transition between equilibria, this additional equilibrium provides a micro-founded mechanism that may help explain previous observational findings on the treatment-access-enhancing effect of insurance.

The study’s most important practical implication concerns the co-payment rate; under the baseline parameterization, insurance loses its power to improve treatment decisions once the co-payment rate exceeds a model-implied threshold of approximately 46.3% (Section 3.3, Table 2). While the exact numerical value of this threshold depends on illustrative parameters that are not directly estimated from data, its existence points to co-payment structure as a policy-relevant design variable that may deserve at least as much attention as premium level—a hypothesis that merits empirical testing before being adopted as a design rule by insurance companies or regulatory bodies.

The findings of this study may also be relevant to veterinary practice, though several caveats apply. Within the model, the availability of the Pareto-superior equilibrium corresponds to an outcome in which comprehensive treatment is recommended by the veterinarian and accepted by the owner when insurance is available. However, because the model does not specify an equilibrium-selection mechanism, it does not imply that veterinarians will necessarily recommend, or owners will necessarily accept, comprehensive treatment more frequently in practice. Whether insurance increases the frequency of such recommendations and treatment acceptance is an empirical question that warrants further investigation. The model also does not establish whether such an outcome would translate into less frequent compromises of treatment protocols or greater scope for evidence-based practice in reality, since clinical appropriateness is not itself a modeled variable [9,11]. More broadly, the expansion of insurance coverage could, as illustrated by the Swedish experience of Egenvall et al. [12], eventually support the development of large-scale insurance databases as a resource for animal health surveillance and epidemiological research in Türkiye; this remains a contextual observation rather than a finding of the present analysis.

Future studies could extend the model to a sequential (Stackelberg-type) game reflecting the realistic clinical order in which the veterinarian moves first; incorporate the insurance company as a third player to offer a full three-sided equilibrium analysis; or calibrate model parameters directly through primary data collection specific to Türkiye (surveys, clinical record analysis). In this direction, the present study aims to add both a theoretical and a policy-oriented layer of analysis to pet insurance research.

## Figures and Tables

**Figure 1:**
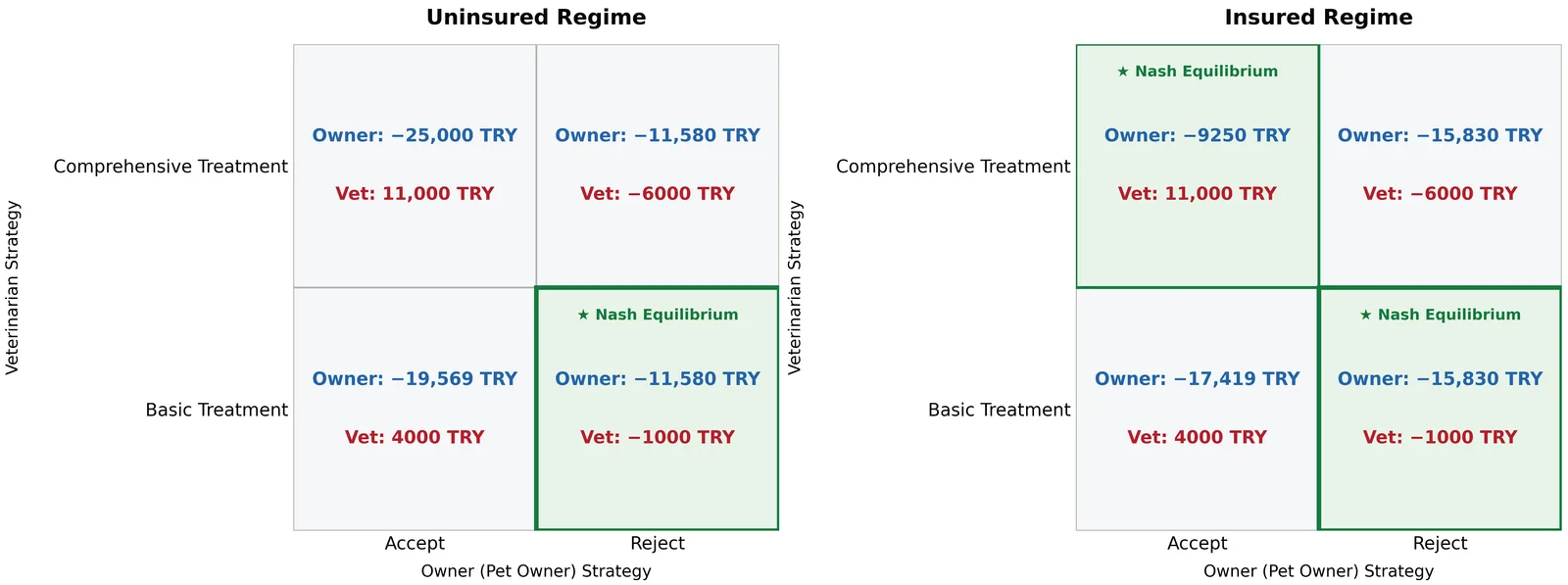
Certainty equivalent (CE) payoff matrices of the owner–veterinarian game (α = 0.0003, *p* = 0.35).

**Figure 3:**
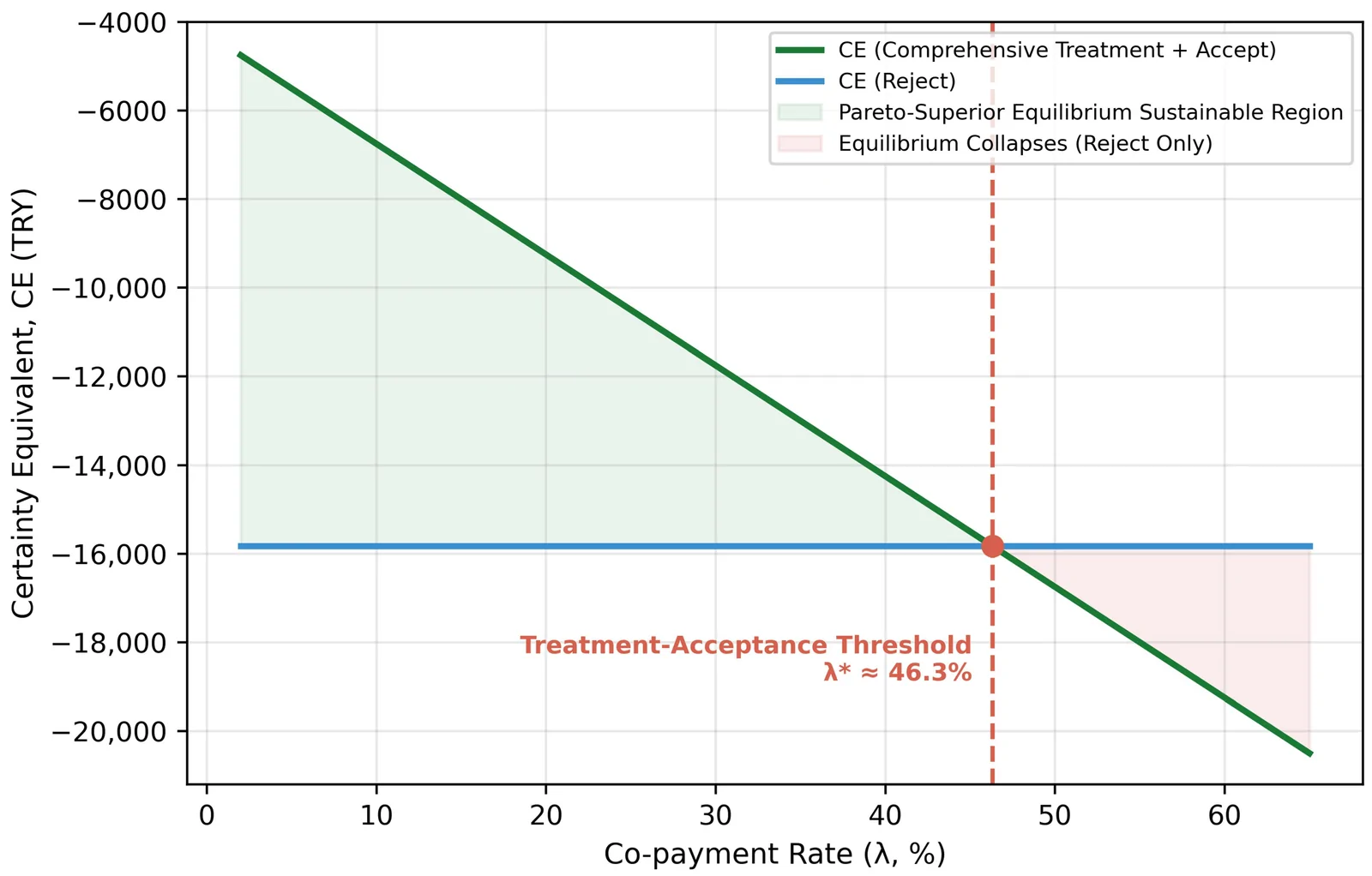
Effect of changes in the co-payment rate on the Pareto-superior equilibrium.

**Figure 4:**
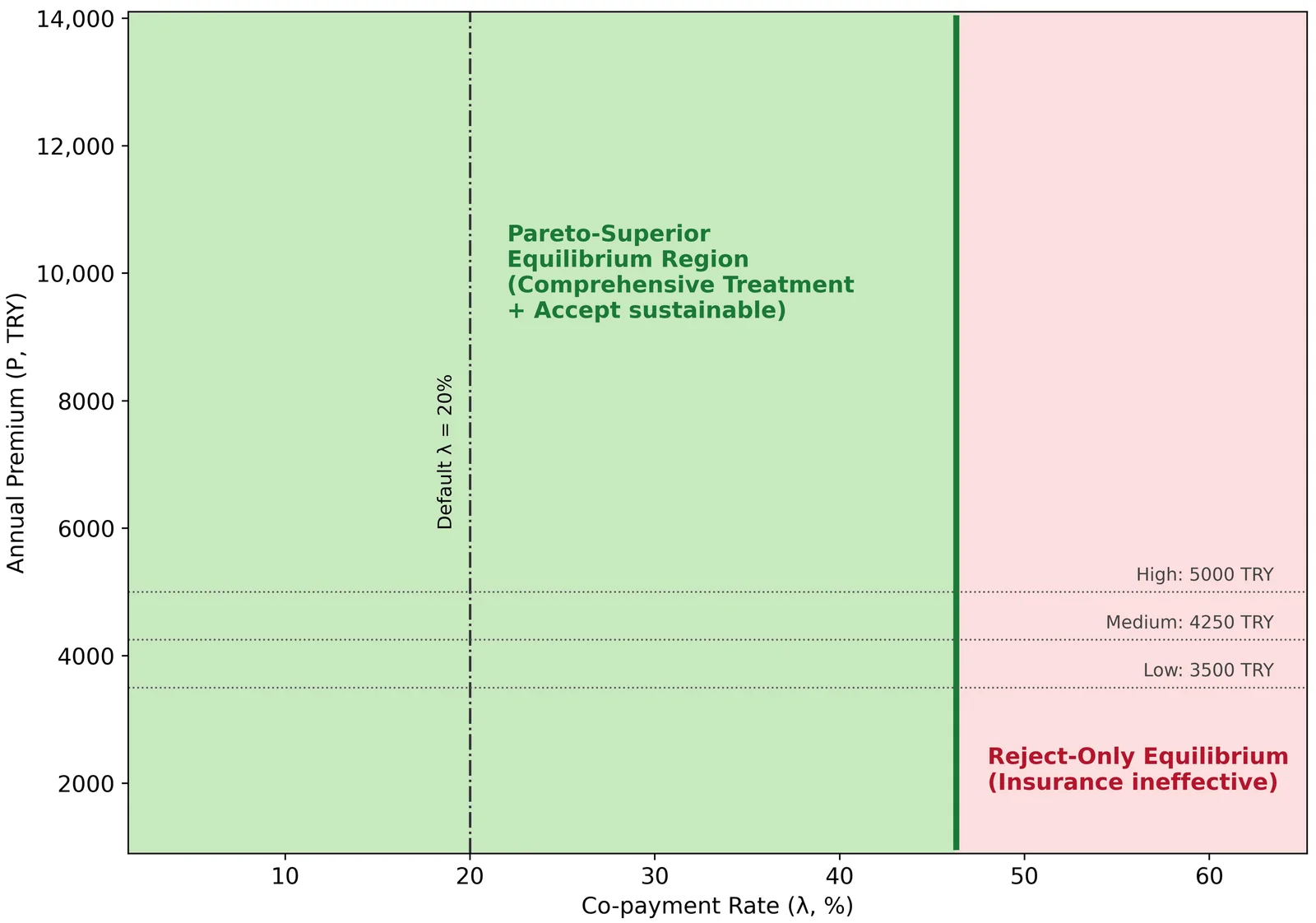
Feasibility region of insurance design: equilibrium map by premium–co-payment combinations.

**Table 1:** Model parameters and sources.

Parameter	Value	Source Type	Rationale/Source
C_T_ (Basic treatment cost)	8000 TRY	Empirical (market data)	Lower bound of basic package coverage range [17]
C_K_ (Comprehensive treatment cost)	25,000 TRY	Empirical (market data)	Upper bound of comprehensive package coverage [17]
P (Annual premium)	3500/4250/5000 TRY	Empirical (market data)	Low/Medium/High premium scenarios [17]
λ (Co-payment rate)	20% (default)	Descriptive benchmark	Simple average of 11 publicly listed co-payment rates/conditions from selected Turkish pet insurance products (approximately 21.36%); 20% is used as the baseline descriptive benchmark [26,27,28,29,30]
p (Probability of severe case)	0.35	Illustrative assumption	Motivated qualitatively by the 60–70% loss ratio in [17]; a loss ratio and a per-case severity probability are conceptually distinct (see Section 2.5 and Limitations)
R_T_, R_K_ (Vet revenue)	6000/20,000 TRY	Derived	~75–80% of treatment cost, based on typical clinical margins
η_T_, η_K_ (Vet operating cost)	2000/9000 TRY	Illustrative assumption	Resource/time cost assumption
ψ_L_, ψ_H_ (Welfare cost of refusal)	500/15,000 TRY	Illustrative assumption	Monetary equivalent of the welfare risk of treatment refusal
κ_T_, κ_K_ (Vet’s reputational/ethical cost)	1000/6000 TRY	Illustrative assumption	Moral cost incurred upon refusal
α (Owner’s baseline risk-aversion coefficient)	0.0003	Illustrative assumption	Selected to produce an observable but moderate risk premium; see sensitivity analysis in Section 3.2.

**Table 2:** Sensitivity of the co-payment threshold (λ*) to model parameters not directly calibrated from market data.

Parameter Varied	Tested Value	λ* (Co-Payment Threshold)
	0.20	39.3%
	0.35 (baseline)	46.3%
p (severe case probability)	0.50	51.0%
	0.65	54.5%
	0.80	57.1%
	10,000	27.4%
	12,500	36.8%
ψ_H_ (welfare cost of refusal, TRY)	15,000 (baseline)	46.3%
	17,500	56.3%
	20,000	66.2%
	3000	46.3%
	4500	46.3%
κ_K_ (vet’s reputational cost, TRY)	6000 (baseline)	46.3%
	7500	46.3%
	9000	46.3%

## Data Availability

No new empirical dataset was generated or analyzed in this study. Model parameters are presented in Table 1, and the sensitivity of key results to illustrative parameters is reported in Table 2; all numerical results can be reproduced using the analytical formulas (Equations (1)–(5)) described in the article.

## Abbreviations

The following abbreviations are used in this manuscript:

CARA	Constant Absolute Risk Aversion
CE	Certainty Equivalent
